# Supplementary material for: The genome sequence of the Brown Heath Robberfly, Tolmerus cingulatus (Fabricius, 1781)
Source: Wellcome Open Res. 2024 Dec 2;9:705. [Version 1] doi: 10.12688/wellcomeopenres.23416.1 (PMC11803401; doi:10.12688/wellcomeopenres.23416.1)
Supplement: Peer review history

# Reviewer report: Reviewer response for version 1

**DOI:** 10.21956/wellcomeopenres.25825.r115114

**Published:** 2024-12-16

**Authors:** Juan Wulff

**Affiliations:** 1 North Carolina State University, Raleigh, USA

The work in general is good and the method used for sequencing quite robust, but description of the same is not detailed, so it is not easily repeatable by other researchers. The manuscript could be a bit more descriptive about some aspects, but I understand that it is more a report than a research paper.

 The main weakness of the study is that the annotation was completed using the Uniprot database without transcriptomes supporting the gene annotation. I believe that the quality of the genome assembly is very good but in terms of gene annotation should be considered a draft genome to be improved in the future.

 Further, It may be useful for future studies if the DNA of bacteria and other microorganisms is provided separately from the genome assembly but all together with an associated ID within the same Bioproject.

 In addition, for future reports, using a scale bar in the pictures could be useful to see the actual specimens’ size.

Are sufficient details of methods and materials provided to allow replication by others?

Partly

Is the rationale for creating the dataset(s) clearly described?

Yes

Are the datasets clearly presented in a useable and accessible format?

Yes

Are the protocols appropriate and is the work technically sound?

Yes

Reviewer Expertise:

Entomology; Genetics.

I confirm that I have read this submission and believe that I have an appropriate level of expertise to confirm that it is of an acceptable scientific standard.

# Reviewer report: Reviewer response for version 1

**DOI:** 10.21956/wellcomeopenres.25825.r115108

**Published:** 2024-12-12

**Authors:** Ruiqi Li

**Affiliations:** 1 University of Colorado Boulder, Boulder, Colorado, USA

The article provides a detailed presentation of the chromosomal-level genome assembly for the Brown Heath Robberfly, *Tolmerus cingulatus*. The genome is well presented, of good-quality but the background could benefit from adding what bilogical or ecological questions could this genome help us to answer. See examples in Li et al .2024.

Are sufficient details of methods and materials provided to allow replication by others?

Yes

Is the rationale for creating the dataset(s) clearly described?

Yes

Are the datasets clearly presented in a useable and accessible format?

Yes

Are the protocols appropriate and is the work technically sound?

Yes

Reviewer Expertise:

Genomics

I confirm that I have read this submission and believe that I have an appropriate level of expertise to confirm that it is of an acceptable scientific standard.

## References

[1] : The genome sequence of the giant clam, Tridacna gigas (Linnaeus, 1758). *Wellcome Open Res* .2024;9: 10.12688/wellcomeopenres.21136.1 145 10.12688/wellcomeopenres.21136.1 38800516 PMC11116938

[2] : The genome sequence of a heart cockle, Fragum fragum (Linnaeus, 1758). *Wellcome Open Res* .2024;9: 10.12688/wellcomeopenres.21134.1 129 10.12688/wellcomeopenres.21134.1 38989474 PMC11234083

[3] : The genome sequence of the heart cockle, Fragum sueziense (Issel, 1869). *Wellcome Open Res* .2024;9: 10.12688/wellcomeopenres.22585.1 366 10.12688/wellcomeopenres.22585.1 39398938 PMC11467648
